# Determinants Affecting the Clinical Implementation of a Molecularly Informed Molecular Tumor Board Recommendation: Experience from a Tertiary Cancer Center

**DOI:** 10.3390/cancers15245892

**Published:** 2023-12-18

**Authors:** Lars Tögel, Christoph Schubart, Sebastian Lettmaier, Clemens Neufert, Juliane Hoyer, Kerstin Wolff, Evgeny A Moskalev, Robert Stöhr, Abbas Agaimy, André Reis, Bernd Wullich, Andreas Mackensen, Marianne Pavel, Matthias W. Beckmann, Arndt Hartmann, Rainer Fietkau, Norbert Meidenbauer, Florian Haller, Silvia Spoerl

**Affiliations:** 1 Institute of Pathology, University Hospital Erlangen, Friedrich-Alexander-Universität Erlangen-Nürnberg (FAU), 91054 Erlangen, Germanyevgeny.moskalev@uk-erlangen.de (E.A.M.);; 2 Comprehensive Cancer Center Erlangen-EMN (CCC ER-EMN), 91054 Erlangen, Germanykerstin.wolff@uk-erlangen.de (K.W.); silvia.spoerl@uk-erlangen.de (S.S.); 3 Bavarian Cancer Research Center (BZKF), 91054 Erlangen, Germany; 4 Department of Radiation Oncology, University Hospital Erlangen, Friedrich-Alexander-Universität Erlangen-Nürnberg (FAU), 91054 Erlangen, Germany; 5 Department of Internal Medicine 1, University Hospital Erlangen, Friedrich-Alexander-Universität Erlangen-Nürnberg (FAU), 91054 Erlangen, Germany; 6 Deutsches Zentrum Immuntherapie (DZI), Friedrich-Alexander-Universität Erlangen-Nürnberg (FAU), 91054 Erlangen, Germany; 7 Institute of Human Genetics, University Hospital Erlangen, Friedrich-Alexander-Universität Erlangen-Nürnberg (FAU), 91054 Erlangen, Germany; 8 Department of Urology and Pediatric Urology, University Hospital Erlangen, Friedrich-Alexander-Universität Erlangen-Nürnberg (FAU), 91054 Erlangen, Germany; 9 Department of Internal Medicine 5, University Hospital Erlangen, Friedrich-Alexander-Universität Erlangen-Nürnberg (FAU), 91054 Erlangen, Germany; 10 Department of Obstetrics and Gynecology, University Hospital Erlangen, Friedrich-Alexander-Universität Erlangen-Nürnberg (FAU), 91054 Erlangen, Germany

**Keywords:** Molecular Tumor Board, clinical decision making, precision medicine, molecular pathology, real-world data, cancer care

## Abstract

**Simple Summary:**

Molecular Tumor Boards (MTBs) utilize comprehensive genomic profiling data to identify and evaluate the therapeutical relevance of cancer-specific genomic vulnerabilities enabling tailored personalized medicine. The present study focused on elucidating factors affecting the clinical translation of MTB recommendations utilizing real-world data in a retrospective analysis. Clinical implementation occurred preferentially when MTB recommendations were of high clinical evidence level. In particular, this was true for recommendations involving PARP, PIK3CA, or IDH1/2 inhibitors. However, we also observed a significant therapeutical benefit of implemented treatments of a low clinical evidence level. This illustrates the limitations of interpreting the therapeutical potential of molecularly informed treatment recommendations solely on their clinical evidence level. The findings of the present study may contribute to the identification of strategies to improve the rate of therapeutical implementation in future studies.

**Abstract:**

Molecular Tumor Boards (MTBs) converge state-of-the-art next-generation sequencing (NGS) methods with the expertise of an interdisciplinary team consisting of clinicians, pathologists, human geneticists, and molecular biologists to provide molecularly informed guidance in clinical decision making to the treating physician. In the present study, we particularly focused on elucidating the factors impacting on the clinical translation of MTB recommendations, utilizing data generated from gene panel mediated comprehensive genomic profiling (CGP) of 554 patients at the MTB of the Comprehensive Cancer Center Erlangen, Germany, during the years 2016 to 2020. A subgroup analysis of cases with available follow-up data (*n* = 332) revealed 139 cases with a molecularly informed MTB recommendation, which was successfully implemented in the clinic in 44 (31.7%) of these cases. Here, the molecularly matched treatment was applied in 45.4% (*n* = 20/44) of cases for ≥6 months and in 25% (*n* = 11/44) of cases for 12 months or longer (median time to treatment failure, TTF: 5 months, min: 1 month, max: 38 months, ongoing at data cut-off). In general, recommendations were preferentially implemented in the clinic when of high (i.e., tier 1) clinical evidence level. In particular, this was the case for MTB recommendations suggesting the application of PARP, PIK3CA, and IDH1/2 inhibitors. The main reason for non-compliance to the MTB recommendation was either the application of non-matched treatment modalities (*n* = 30)/stable disease (*n* = 7), or deteriorating patient condition (*n* = 22)/death of patient (*n* = 9). In summary, this study provides an insight into the factors affecting the clinical implementation of molecularly informed MTB recommendations, and careful considerations of these factors may guide future processes of clinical decision making.

## 1. Introduction

Next-generation sequencing (NGS)-mediated comprehensive genomic profiling (CGP) provides a broadly available and relatively cost-effective method to screen cancer tissues for targetable genetic alterations on a molecular level. The frequency of clinically relevant alterations varies between entities, and also between different study cohorts, with approximately 7.5–57% of screened cancer patients being eligible for a targeted therapy based on molecular alterations [1,2,3,4]. CGP is available to cancer patients within different settings, either by commercial providers [5] or embedded within an academic clinical context [6,7,8]. For the latter approach, Molecular Tumor Boards (MTBs) have been implemented in most academic tertiary clinical centers in Germany within the last few years, to provide CGP for cancer patients within a framework of clinical interpretation of molecular alterations [9,10,11,12,13]. The clinical benefit for cancer patients who have undergone CGP in the framework of an MTB has been repeatedly demonstrated [6,7,8,14]. Of note, however, are certain disparities, such as the utilization of different gene panel contents or variant annotation pipelines hampering inter-institutional comparisons. To account for these disparities and to ensure harmonized molecular testing and clinical interpretation procedures throughout local MTBs, different regional and national networks are currently being established, such as the German Cancer Consortium (DKTK), Centers for Personalized Medicine (ZPM), Bavarian Cancer Research Center (BZKF), and genomeDE. Regarding the increasing demand for CGP by cancer patients and their treating physicians, MTBs are on their way to becoming the routine clinical standard for cancer patients with advanced disease. Of note, key prerequisites determine the successful implementation of a local MTB, which include the on-site availability of NGS technology, the applicability of available tumor material, cost effectiveness, the availability of clinical expertise in the local MTB, and the regional availability of clinical trial opportunities. Currently, another limiting factor for successful implementation of an MTB represents the outreach of academic tertiary clinical centers. For instance, 1.374 patients with advanced cancers had been discussed within a regional MTB framework at four academic tertiary clinical centers in a two-year time frame covering the federal state of Bavaria, Germany [13]. At a comparable time frame, however, the absolute number of cancer patients in Bavaria comprised ~130.00 patients [15]; thus, only 1% of all cancer patients had been sequenced and discussed at four local MTBs. Although not all cancer patients would qualify for CGP according to localized cancer disease and/or standard therapy options, it can be estimated that the number of cancer patients at each academic MTB site will significantly increase in the coming years. From an ethical point of view, each patient with advanced cancer should have the same chance to receive CGP of his/her cancer tissue to screen for targetable molecular alterations. However, this will dramatically increase the number of patients to be analyzed and discussed at local MTBs. Therefore, it is of high importance to identify factors contributing to successful implementation of therapeutically relevant MTB recommendations by treating physicians. Currently, there is a significant difference between the percentage of MTB patients with therapeutic recommendations and MTB patients who in fact receive the recommended therapy [6,7,8,9,10,11,12,13,14]. The current study analyzes genomic and clinical follow-up data generated in the context of an MTB of an academic tertiary clinical comprehensive cancer center, to identify factors correlative to the adherence to MTB therapeutic recommendations by the treating physician in a real-world scenario.

## 2. Materials and Methods

### 2.1. Study Cohort

A formalin-fixed, paraffin-embedded (FFPE) specimen of 590 consecutive cancer patients were collected and subjected to molecular diagnostic testing at the Institute of Pathology of the University Hospital Erlangen, Germany, between May 2016 and the end of December 2020. Human patient samples were used in accordance with ethical guidelines for the use of retrospective tissue samples, and ethical review and approval were obtained from the local ethics committee of the Friedrich-Alexander University Erlangen-Nuremberg (ethics committee statements 100_17 B from 7 April 2017, addendum from 27 July 2021).

### 2.2. The Molecular Tumor Board

The multi-disciplinary MTB at the Comprehensive Cancer Center Erlangen-EMN (Europäische Metropolregion Nürnberg) was held bi-weekly until the end of the year 2020 and then changed to a weekly meeting due to increasing patient numbers. Internal or external oncologists and treating physicians were able to ask for CGP. Before MTB registration, cases were discussed at the respective Organ Tumor Board, and referral to the MTB was concluded. Patients fulfilling any of the following criteria were eligible for MTB enrollment: (a) advanced tumor disease and expected end of standard therapy within the next six months; (b) patients suffering from a cancer of unknown primary syndrome (CUP); or (c) patients harboring a tumor disease with a very atypical course of disease or combination of several tumors. The most recent tumor material was used for comprehensive molecular profiling. The core panel of the MTB consisted of at least one representative of the following disciplines: oncology, pathology, bioinformatics, molecular biology, or human genetics, as well as the treating oncologist/physician.

### 2.3. Next-Generation Sequencing

An H&E stain of formalin-fixed, paraffin-embedded cancer tissue was used for routine tumor diagnosis and for tumor cell content estimation. After microdissection of the tumor tissue, both DNA and RNA were isolated using standard techniques (Kit, Promega Maxwell). Initially, only the DNA content was genetically screened utilizing a 160-gene panel (Comprehensive Cancer Panel, Qiagen, Hilden, Germany), as published previously [16]. From 2018 onwards, DNA and RNA were analyzed using a 170-gene panel based on hybrid-capture enrichment (TruSight Tumor 170, TST170, Illumina, San Diego, CA, USA) according to the manufacturer’s instructions and as described elsewhere [17]. Obtained sequences were aligned with the reference sequence hg19. Alterations were annotated using VariantStudio (V3.0, Illumina) or the open-source tool Ensembl Variant Effect Predictor [18] and described using standard HGVS nomenclature [19]. In addition, changes in gene copy numbers were determined by the CRAFT copy number variant caller (v1.0.0.12) algorithm incorporated in the TST170 application. On the RNA level, high-confidence gene fusions or alternative splice-variants were directly obtained by using the TST170 application on the BaseSpace Sequence Hub (Illumina).

### 2.4. Variant Classification

For the functional interpretation and classification of non-synonymous variants on DNA-level as well as gene fusions on RNA-level, the following databases were used: ClinVar, VarSome, cbioPortal, Molecular Tumor Board Portal, LOVD3, Mitelman Database, and ChimerDB 3.0 database [20,21,22,23,24,25,26,27,28].

### 2.5. Evidence Levels for Biomarker Stratification

The evidence level classification of variants was performed according to the evidence levels defined by Nationales Centrum für Tumorerkrankungen (NCT) and Deutsches Konsortium für Translationale Krebsforschung (DKTK) [29,30]. According to this scheme, the following NCT evidence levels were applied to genetic alterations: M1 (biomarker for the same tumor entity, with further subdivision according to study design M1A–M1C); M2 (biomarker for another tumor entity, with further subdivision according to study design M2A–M2C); M3 (biomarker based on preclinical in vitro/in vivo data); M4 (biomarker based on biological rationale). 

### 2.6. Data Visualization 

GraphPad Prism v9.5.1 (GraphPad Software, San Diego, CA, USA) and Arrriba v.2.4.0 [31] were used for statistical analysis and graph generation.

## 3. Results

### 3.1. Study Cohort, Patient Demographics, and Molecular Findings

**Figure 1:**
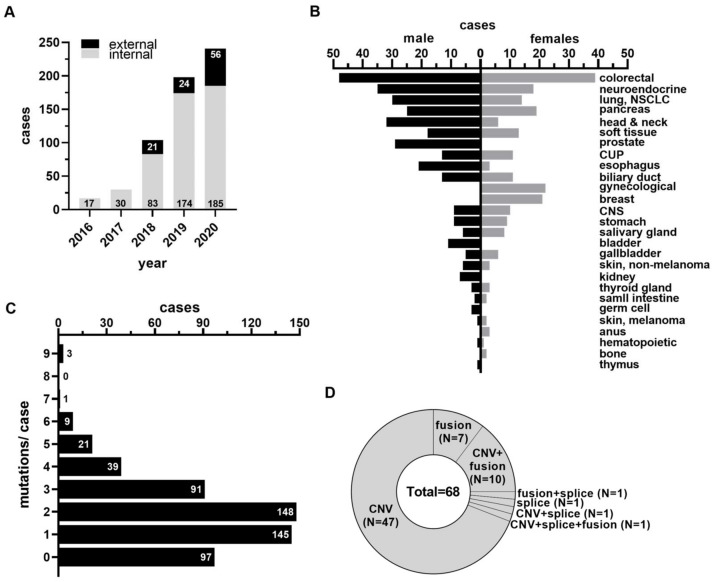
Basic MTB cohort characteristics, patient demographics, and molecular findings. (**A**) Bar graph representing the numbers of MTB case presentations per year. Black bar fractions symbolize the proportion of external referrals to the MTB. (**B**) Number of cases dichotomized by patient gender and site of primary, rank ordered by total case numbers. (**C**) Number of concurrent mutations (SNVs, MNVs, small insertions/deletions) per case. (**D**) Cases without mutations but with structural variants (CNVs, fusion, splice variants).

The interdisciplinary Molecular Tumor Board (MTB) at the Comprehensive Cancer Center Erlangen was initiated in May 2016 with the particular aim to provide elaborated guidance in clinical decision making to the treating physician of patients with advanced malignant disease or with cancers of atypical presentation. The core team of the MTB consists of pathologists, oncologists and other clinicians, molecular biologists, as well as genetics counselors and is complemented by the respective treating physician or medical doctor who asked for molecular testing. The present study describes the findings of the initial patient cohort presented at the MTB between May 2016 and the end of December 2020. Within this time frame, patient numbers constantly increased on a yearly basis, starting with 17 patients enrolled in 2016, and peaking with 241 patients in the year 2020 (Figure 1A).

Likewise, the number of external patients referred to the MTB from associated clinics and physicians from outside the University Hospital of Erlangen gradually increased within this time frame, peaking in 2020 with a total of 56 cases (Figure 1A). In total, molecular testing inquiries for 590 (internal/external: 489/101) patients were received within this period, of which 554 (93.9%) underwent successful next-generation sequencing (NGS) processing (Table 1). 

The main reasons for drop-out were insufficient amount/quality of tumor material or low-quality sequencing results. At the time of MTB discussion, most patients (79.8%) presented with advanced, metastatic disease and had received at least one previous line of therapy (83.1% of all patients, Table 1). Male patients (59.2%) were more prevalent in this study cohort than female patients (40.8%), probably owing to the relatively low numbers of gynecological and breast cancer cases (Table 1, Figure 1B). On average, male patients were slightly older (median: 63 years, range: 19–88 years) than female patients (median: 57 years, range 16–88 years) (Table 1). The median time from initial cancer diagnosis to presentation at MTB was 19 months (Table 1). Only two patients with hematological malignancies (1x acute myelogenous leukemia, 1x multiple myeloma) were presented at the MTB, whereas all other patients had solid cancer types (Table 1, Figure 1B). Colorectal cancer was the most prevalent entity (*n* = 87, 15.7% of cases), followed by neuroendocrine neoplasms (9.6%), and cancers of lung (7.9%), pancreas (7.9%), head and neck region (6.9%), soft tissue (6%), and prostate (5.2%) (Table 1, Figure 1B). Neuroendocrine neoplasms (NEN) are a heterogenous but rare group of tumors accounting for approximately 2% of all newly diagnosed cancers. A comprehensive molecular and clinical analysis of an extended NEN cohort will be conducted in a separate study (doctoral thesis of L. Bindemann, work in progress). While most of the cancer entities were more or less equally distributed between male and female patients, the proportion of cancers of the head and neck, esophagus, bladder, kidney, and germ cell were higher in males compared to their prevalence in female patients, which is in accordance with their clinical predominance in men than in women (Figure 1B). It is worth mentioning that the study cohort also included a significant number of cases with cancer of unknown primary (CUP, *n* = 26, 4.7% of all cases). CUPs represent metastasized tumors for which histopathological features yielded insufficient information for the identification of tissue of the primary origin, accounting for 3–5% of all diagnosed cancers [32]. Median time between CUP diagnosis and MTB inclusion was 3 months in the present study. NGS-mediated CGP failed to identify any mutation in three cases, allowing no further conclusion on cancer origin. In 22 cases, however, NGS-mediated CGP detected at least one mutation, which enabled CUP re-classification to the cancer of origin in 12 cases (Figure S1). This analysis highlights that, in addition to deciphering predictive biomarkers for treatment stratification, CGP for primary identification represents yet another aim of the MTB and further stresses its powerful diagnostic capabilities. 

The functional consequences of genomic alterations identified by gene panel mediated CGP were determined utilizing available information from several databases and the literature. For the sake of biomarker stratification, a pathogenicity score was assigned to each identified variant and the clinical evidence level and therapeutical relevance of likely pathogenic or pathogenic non-synonymous variants was then evaluated in the context of the respective tumor entity in the interdisciplinary MTB according to a classification scheme described previously [29,30]. Non-synonymous variants without proven functional impact (variants of uncertain significance, VUS) were recorded but were excluded from further analysis because of their unknown clinical relevance. The CGP of 554 cases revealed 457 (82.5%) cancer cases with at least one likely pathogenic or pathogenic mutation (i.e., single nucleotide variant, SNV; multiple nucleotide variant, MNV; short insertions/deletions, Figure S2A). For most of the tumors, one (*n* = 148) or two concurrent (*n* = 145) likely pathogenic/pathogenic mutations were detected (Figure 1C). In a subgroup, CGP revealed three (*n* = 91), four (*n* = 39), five (*n* = 22), or more than five (*n* = 12) concurrent likely pathogenic/pathogenic mutations per patient, respectively (Figure 1C). Of note, in some of the aforementioned cases, gene panel-mediated CPG also revealed more complex genetic aberrations, i.e., structural variants including gene copy number alterations (copy number variants, CNV), gene fusions, or splice alterations in addition to likely pathogenic/pathogenic mutations (Figure S2B, Figure S2C, Table S1). In 97 cases, gene panel-mediated CPG detected no likely pathogenic/pathogenic mutations (Figure 1C). However, the majority of these cases (*n* = 68) displayed CNVs, gene fusions, splice alterations, or a combination thereof (Figure 1D). Gene panel-mediated CGP was unable to identify any of the aforementioned genetic alterations (mutations and/or structural variants) in only a fraction of cancer cases (*n* = 29; 5.2%). On the basis of gene panel-mediated CGP data, a molecularly informed treatment recommendation was defined at the interdisciplinary MTB and communicated to the treating clinician. The factors determining the clinical implementation of the MTB recommendation and the potential therapeutical benefit resulting from matched-therapy application is the subject of the current study and will be discussed in the following sections.

### 3.2. Implementation of Molecular Tumor Board Recommendations

**Figure 2:**
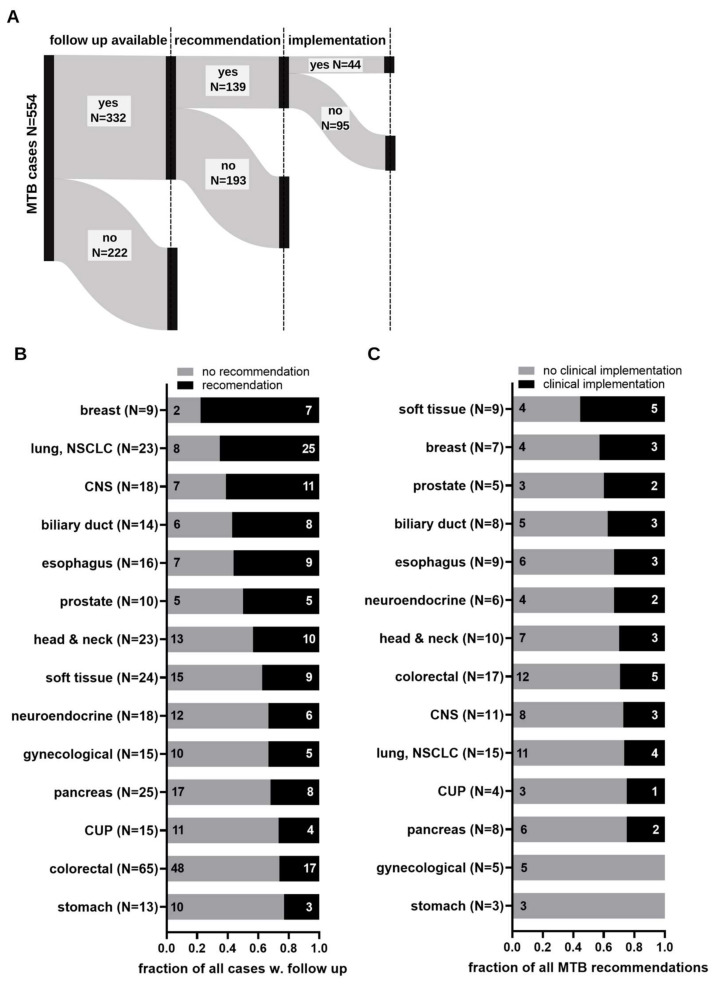
Clinical follow-up and implementation of MTB recommendations. (**A**) Summary of cases with clinical follow-up data, recommendation, and implementation status. (**B**) Proportion of cases without (grey fraction) and with (black fraction) MTB recommendations dichotomized by organ of primary. Numbers indicate the quantity of each fraction. Only entities with data for 9 or more cases are shown. (**C**) Proportion of clinical implementation of MTB recommendations per entity from (**B**). Black fractions indicate the proportion of recommendations with clinical translation. Numbers indicate the quantity of cases of implemented recommendation.

To elucidate the impact of gene panel-mediated CGP on therapeutical decision making, we retrospectively acquired individual clinical follow-up data from cases presented at the MTB and first interrogated the level of recommendation implementation. Of 554 discussed cases, clinical follow-up was available for 332 (59.9%) patients, while 222 (40.1%) patients were lost to follow-up (Figure 2, left column). 

For 139 (41.9%) of 332 patients with available clinical follow-up data, gene panel-mediated CGP led to an informed therapy recommendation, while the remaining 193 (58.1%) patients received no therapeutic recommendation (Figure 2A, middle column). Overall, 44 (31.7%) of 139 patients with therapy recommendation received the suggested treatment regimen, whereas 95 (68.3%) patients did not (Figure 2, last column). To decipher the subgroup of patients with a high potential for clinical benefit from MTB inclusion, a more granular analysis of tumor entity level was conducted within the group of 332 cases with clinical follow-up. In a first analysis, we aimed to identify cancer types with elevated numbers of recommendations and, to ascertain meaningful results, focused solely on entities with a sufficient (≥9) number of cases with follow-up data. This analysis revealed breast cancer as the entity with the most MTB treatment recommendations per number of cases (*n* = 7/9, 77.8%), followed by cancers of the lung (NSCLC *n* = 15/23, 65.2%), CNS (*n* = 11/18, 65.2%), biliary duct (*n* = 8/14, 57.1%), esophagus (N = 9/16, 56.3%), prostate (N = 5/10, 50%), head and neck (*n* = 10/23, 43.5%), soft tissue (*n* = 9/24, 37.5%), neuroendocrine origin (*n* = 6/18, 33.3%), gynecological origin (*n* = 5/15, 33.3%), pancreas (*n* = 8/25, 32%), CUP (*n* = 4/15, 26.7%), colorectal (*n* = 17/65, 26.2%), and stomach (*n* = 3/13, 23.1%) (Figure 2B). Next, we determined the entities with the highest rate of clinical implementation of MTB recommendations within the aforementioned entities and identified soft-tissue tumors as the entity with the highest rate of adherence to of MTB recommendations (Figure 2C). Here, 55.6% (*n* = 5/9) of all treatment recommendations were followed. The high rate of compliance may be explained, at least to some extent, by the low numbers of available approved treatment options for this disease. Breast cancer ranked second in terms of implementation rates, with 3 out of 7 (42.9%) recommendation adherence, followed by cancers of the prostate (*n* = 2/5, 40%), biliary duct (*n* = 3/8, 37.5%), esophagus (*n* = 3/9, 33.3%), neuroendocrine origin (2/6, 33.3%), head and neck (*n* = 3/10, 30%), colorectal (*n* = 5/17, 29.4%), CNS (*n* = 3/11, 27.3%) NSCLC (*n* = 4/15, 26.7%), pancreas (*n* = 2/8, 25%), and CUP (*n* = 1/4, 25%). No clinical MTB recommendation implementation was observed for stomach or gynecological cancers (Figure 2C). In summary, gene panel-mediated CPG resulted in a high proportion of MTB cases with an NGS analysis-informed treatment recommendation (approx. 40% of all cases with available follow-up). The clinical translation of MTB recommendations was observed in a significant proportion of these recommendations (*n* = 44, 31.7%); however, the implementation rate differed between entities. Given the heterogeneity of clinical translation rates of MTB recommendations, we interrogated the underlaying factors of these disparities in the following analysis.

### 3.3. Determinants Impacting on Clinical Implementation of Molecular Tumor Board Recommendations

**Figure 3:**
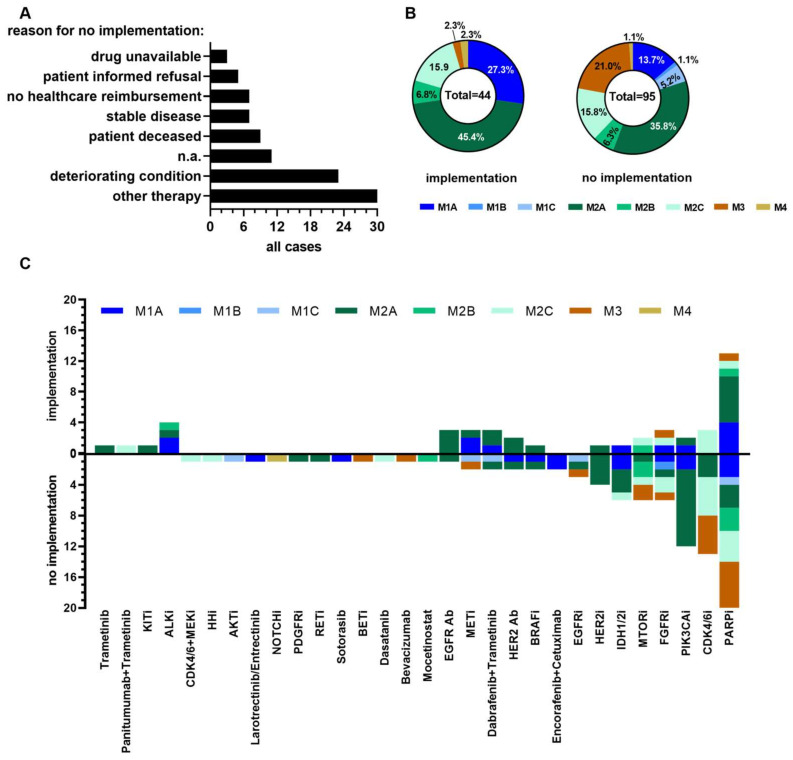
Clinical follow-up and implementation of MTB recommendations. (**A**) Bar graph represents the number of cases that failed clinical translation stratified by particular reason for failure. (**B**) Granular analysis of MTB recommendation implementation on clinical evidence level. Proportions of individual level of evidence of recommendation among cases with (left donut chart) and without clinical translation (right donut chart). (**C**) CGP-informed, molecularly matched treatment recommendations stratified by clinical translation status and level of evidence. Shown are the clinical translation (upper bar graph) or non-implementation (lower bar graph) of recommendations for particular drug classes/specific inhibitors.

In order to decipher factors impeding on the clinical translation of MTB recommendations and to identify patient subgroups who are likely to receive the molecularly matched treatment regimen, we first interrogated the main reasons for the failure of the clinical translation within the study cohort. This analysis revealed that adherence to non-molecularly matched treatment modalities (e.g., chemotherapy or radiation therapy within cancer therapy guidelines, *n* = 30) and deteriorating patient condition (*n* = 22) represented the main reasons for the failure of the MTB recommendation translation. The less prevalent, i.e., death of patient (*n* = 9) and no disease progression at time of data cut-off (stable disease, *n* = 7, see Figure 3A), resulted in the failure of the MTB recommendation implementation.

In the remaining cases, no health insurance reimbursement of proposed treatment costs (*n* = 7), informed refusal by the patient (*n* = 6), or drug unavailability at time of MTB (*n* = 3) precluded clinical translation of MTB recommendation. In 11 cases, the reasons for non-adherence were not available (data summarized in Figure 3A). Due to low case numbers, a more granular analysis on tumor type level was not conclusively applicable; nevertheless, at least for some entities, a trend was observed. For instance, the main reason for non-adherence to MTB recommendations in CNS and colorectal cases was the deteriorating health condition/death of the patient, whereas in the context of soft tissue, breast, prostate, pancreas, and lung cancer, the sustained clinical benefit of current, non-molecularly matched therapy/no disease progression (stable disease) eliminated the absolute necessity for a change in treatment regimen at the time of MTB admission (summarized in Table 2). 

In the next step of the analysis, we determined whether the clinical evidence level impinged on the clinical translation of a particular molecularly informed MTB recommendation. Figure 3B shows the summary of clinical evidence levels of MTB recommendations that have been implemented in the treatment of patients (left diagram) and that have not been clinically translated (right diagram). Approximately one-third (27.3%) of implemented MTB recommendations were of the highest clinical evidence level, i.e., recommendations based on the presence of a predictive biomarker for a drug or treatment modality with proven clinical effectiveness in the same entity (evidence level M1A, M1B, or M1C, respectively, Figure 3B left panel, blue slice and Table 3). 

**Figure 4:**
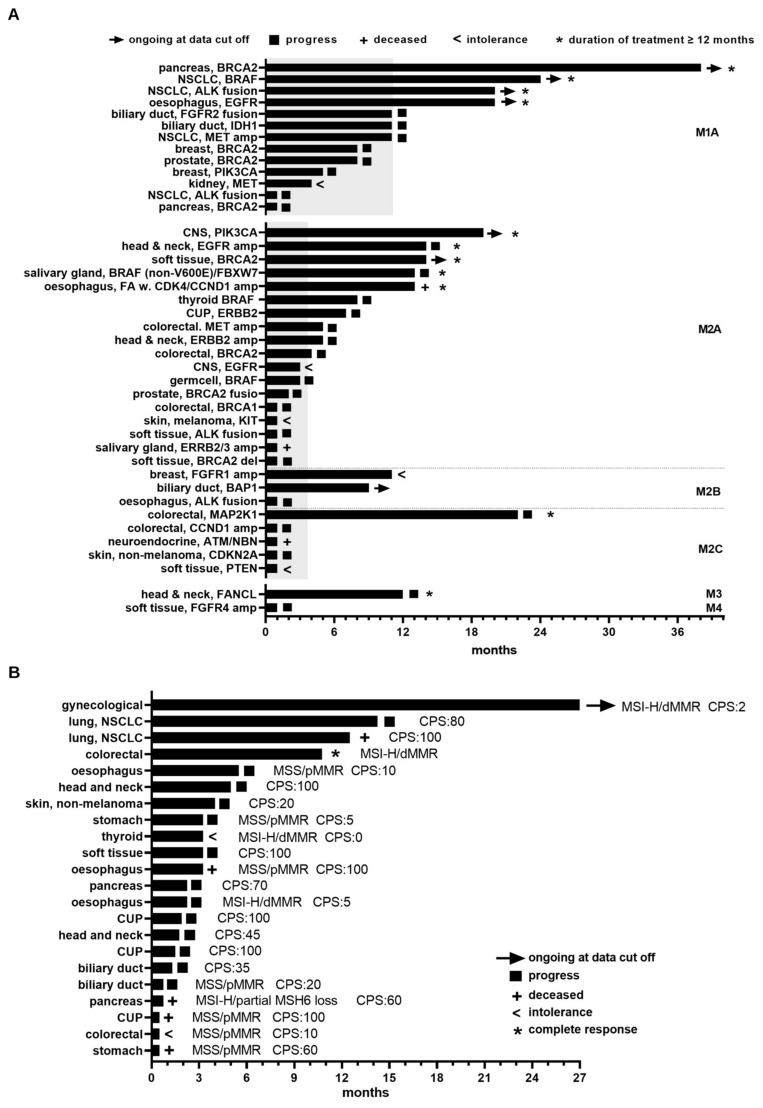
Trajectories of individual treatment responses over time. Bars represent the time to treatment failure (TTF) either due to progress, as indicated by squares, and patient’s death (as indicated by “+”), or until development of intolerance to therapeutic modality (as indicated by “<”). Arrow heads depict ongoing treatments beyond time of data-cut off. Time of treatment is shown in months on the *x*-axis. Entity and the corresponding gene alteration leading to the MTB treatment recommendation formulation are shown on the *y*-axis. (**A**) Swimmer blot displays available data of 41 patients with CPG-informed molecularly matched MTB treatment recommendations. Bars indicate the (TTF) in months and are dichotomized by evidence level. Grey rectangles indicate the median TTF for either M1 (upper panel) or M2 (middle panel) level recommendations. Three patients received molecularly matched therapy less for one month and, therefore, are excluded from the figure. (**B**) Swimmer blot of 22 patients receiving ICI. Abbreviations: MSS = microsatellite stable; MSI-H = microsatellite instability high; dMMR = mismatch repair gene deficiency; CPS = combined positivity score.

The majority (68.1%) of MTB recommendations with clinical translation, however, represented recommendations for tier 2 evidence level therapeutic interventions, i.e., recommendations based on the presence of a predictive biomarker for a drug or treatment modality with proven clinical effectiveness in a histopathologically different entity (evidence level M2A, M2B, or M2C, respectively, Figure 3B, left panel, green slices, and Table 3). Interestingly, among MTB recommendations with clinical adherence were also two cases with actionable alterations with a low clinical evidence classification based solely on pre-clinical data (evidence level M3) or biological rationale (evidence level M4). These cases included a head and neck cancer patient with a FANCL p.Tyr111Cys missense mutation who received the PARP-inhibitor olaparib for 12 months until progress, and a patient with a FGFR4 amplified (gene copy number, GCN:4.5) soft-tissue tumor who received the FGFR-inhibitor ponatinib for 1 month until progress (Figure 4A). In contrast, the proportion of the M3/M4 lowest evidence level was significantly higher (22.1% vs. 4.6%) in the subgroup of patients without clinical implementation of MTB recommendations (Figure 3B, left panel, and summarized in Table S2). About half (*n* = 55 of 95 cases, 57.9%) of MTB recommendations that failed clinical translation represented recommendations for tier 2 (M2A/B/C) evidence level therapeutic interventions (Figure 3B, left panel, Table S2). For most of the cases, either deteriorating patient condition/death of patient precluded recommendation implementation (*n* = 18), or the sustained clinical benefit of current therapy/no disease progression (stable disease) precluded the implementation of the MTB recommendation (*n* = 21). In a further 10 cases, treatment costs were not covered by the health insurance company (*n* = 4), the drug was unavailable (*n* = 2), or the patient declared an informed refusal to undergo the proposed treatment (*n* = 4). Among MTB recommendations that failed clinical implementation were also 19 (20%) tier 1 recommendations (Figure 3B, blue slices. In most of these cases, reasons for non-adherence were a lack of disease progression (stable disease, SD) under current therapy/response to a non-matched anti-cancer treatment (*n* = 7) at time of MTB inclusion, or deteriorating patient condition/patient’s death precluded implementation of tier 1 recommendation (*n* = 6). The latter clearly indicates that an earlier MTB inclusion would have been advantageous in these particular cases. Cancers of the biliary duct, lung (NSCLC), and breast represented the entities with the highest proportion of non-adherence to tier 1 recommendations. With regard to the biliary duct cancer cases, reasons for non-adherence to tier 1 recommendations were deteriorating patient condition (ivosidenib, IDH1 p.Arg132Cys mutation), informed refusal by the patient (pemigatinib, FGFR2 p.Glu566Gly/p.Cys383Arg mutation), and no cost reimbursement by the health care provider (ivosidenib, IDH2 p.Arg172Lys mutation). For NSCLC, recommendations comprised larotrectinib/entrectinib (unconventional SLC28A3::NTRK2 gene fusion), capmatinib (MET amplification, GCN: 16.7), afatinib (RBPMS::NRG1 gene fusion), and dabrafinib/trametinib in the case of a BRAF p.Val600Glu, and a non-V600E BRAF mutated tumor, respectively. In three out of five of these cases, the reasons for non-adherence to MTB recommendations were unknown, in the cases of a NSCLC with NTRK fusion or with BRAF p.Val600Glu mutation; however, stable disease under non-matched therapy precluded MTB recommendation implementation. Similar, ongoing therapeutical benefit was observed for both breast cancer cases during non-matched treatment modalities; therefore, MTB recommendations, i.e., PARP-inhibitor olaparib for the BRCA2 p.Asn433GlnfsTer18/ATM p.Ser2259TyrfsTer13/CHEK2 p.Ile188Thr-mutated cancer and alpelisib for the PIK3CA p.His1047Arg-mutated cancer, were not clinically translated until time of data cut-off. As mentioned earlier, stomach and gynecological cancers represented the only entities without any clinical translation of MTB recommendations (Figure 2C). Of note, however, the group of stomach and gynecological cancers also included cases with MTB tier 1 recommendations for high-potential therapeutical interventions. A closer examination of these cases revealed that clinical implementation was not considered due to worsened health condition of the patient with a CHEK2 p.Thr410MetfsTer15-mutated high-grade ovarian serous adenocarcinoma (recommendation: PARP inhibitor) and due to the application of immune checkpoint inhibitor therapy in the context of a significantly elevated combined positivity score (CPS) of 75 in an ERBB2-amplified (GCN: 25.3) stomach cancer (MTB recommendation: trastuzumab).

Since some of the aforementioned inhibitors represent approved treatment modalities in particular entities (e.g., olaparib and alpelisib for breast cancer), we next interrogated whether recommendations for certain inhibitors were preferentially implemented and therefore dichotomized recommendations for certain drug classes according to their clinical translation and clinical evidence status. The majority of MTB recommendations included specific inhibitors of PARP (*n* = 33), CDK4/6 (*n* = 16), PIK3CA (*n* = 14), FGFR (*n* = 9), ERBB2/HER2 (*n* = 9; 4x antibodies plus 5x small molecular inhibitors), MTOR (*n* = 8), IDH1/2 (*n* = 7), MET (*n* = 5), and the combination of dabrafenib + trametinib (*n* = 5), and recommendations were adhered to in 39.4%, 18.8%, 14.3%, 33.3%, 33.3%, 25%, 14,3%, 60%, and 60% of cases, respectively (Figure 3C). High clinical evidence level (i.e., tier 1: M1A/B/C) recommendations for PARP inhibitors were preferentially implemented in the clinic, as opposed to tier 2/3 (i.e., M2A/B/C and M3) recommendations. Here, 50% (*n* = 4) of tier 1 recommendations were clinically translated, in contrast to only 9 (36.0%) of tier 2/3 recommendations (Figure 3C). Likewise, in the case of IDH1/2 inhibitors, the data suggested that tier 2 evidence level MTB recommendations are prone to fail clinical translation, since clinical adherence occurred in only one of three cases with M1A recommendation but not in all other cases with tier 2 evidence level. This was similar for PIK3CA inhibitor recommendations, only one of the eight M2A clinical evidence level MTB recommendations was translated into the clinic, indicating that recommendations below tier 1 evidence level are less preferentially put into clinical practice. No apparent role of evidence level in decision making was observed for recommendations comprising inhibitors for CDK4/6, FGFR, MTOR, HER2, MET, and the combination of dabrafenib/trametinib (Figure 3C). Of note, all but two recommendations for therapies with M3 evidence level, i.e., olaparib for the treatment of a head and neck cancer with FANCL mutation and ponatinib for the treatment of a FGFR4-amplified soft tissue tumor, failed clinical translation. In contrast, in the context of ALK gene fusion positive cancers (2x NSCLC, 1x esophagus, and 1x soft tissue), all recommendations (*n* = 4) for ALK inhibitors were clinically translated, irrespective of the level of evidence. In summary, this analysis revealed that the main reasons for clinical implementation failures were either (i) the implementation of non-matched treatment modalities, (ii) deteriorating patient condition, or (iii) death of patient. The latter two suggest an earlier MTB inclusion would have been beneficial in these particular cases. Recommendations for specific inhibitors of PARP, PIK3CA, and IDH1/2 are preferentially translated in the context of high (tier 1) clinical evidence, whereas for other inhibitors, no bias for level of evidence was observed.

### 3.4. Clinical Benefit Arising from MTB Recommendations

As outlined above, CGP-informed MTB recommendations led to clinical application of a molecularly matched targeted therapy in 44 cases. In 3 cases, treatment was applied for less than a month because the patient deceased (non-melanoma skin cancer with a double CDKN2A nonsense mutation, therapy: palbociclib) or experienced severe side effects after initial drug application (CNS with non-V600E BRAF and NRAS mutation, therapy: dabrafenib plus trametinib; TSC2-mutated neuroendocrine carcinoma, therapy: everolimus). Therefore, these cases were excluded from subsequent analysis. The remaining 41 cases with molecularly informed therapies displayed a median time to treatment failure (TTF) of 5 months. Among these cases, 13 patients received a therapy based on a tier 1 (M1A) clinical evidence level MTB recommendation, which resulted in a comparably high median TTF of 11 months (min: 1 month, max: 38 months, ongoing at data cut-off, Figure 4A, top panel). 

In 26 cases, patients received a treatment regimen based on tier 2 (as summarized in M2A/B/C, Figure 4A, middle column) clinical evidence level MTB recommendations for a median TTF of 3.5 months (min: 1 month, max: 22 months, ongoing at data cut-off). As outlined before, only two patients with low clinical evidence MTB recommendations (M3/M4) received the molecularly informed treatment (Figure 4A, lower panel). Within these three groups, eleven patients (25%) showed an exceptionally long response to matched targeted therapy for 12 months or longer (4x M1A, 5x M2A, 1x M2C, and 1x M3 clinical evidence level, as indicated by an asterisk in Figure 4A). Three of these patients had an alteration in a gene involved in DNA damage repair (2x BRCA2 and 1x FANCL mutation), and one patient who was diagnosed with Fanconi anemia (FA) before MTB inclusion had a CDK4/CCND1 co-amplification, whereas the majority (seven patients) displayed activating alterations of protein kinases (Figure 4A). The patients with BRCA2 mutation (1x pancreas, 1x soft tissue sarcoma) received olaparib mono-therapy (plus pembrolizumab after 3 months in the case of soft tissue sarcoma) and continued treatment at time of data cut-off. The patient with the FANCL (head and neck cancer) received olaparib and experienced a TTF for 12 months until progress, whereas the FA patient with CDK4/CCND1 amplification deceased after 13 months, with the first treatment being olaparib (6 months) and then palbociclib (7 months). The patients with protein kinase alteration were treated as follows: trametinib plus dabrafenib (BRAF mutated NSCLC, for 24 months, ongoing at data cut-off); panitumumab + trametinib (colorectal cancer with MAP2K1 mutation, 22 months, progress); alectinib (NSCLC, ALK fusion, 20 months, ongoing at data cut-off); osimertinib (esophagus, EGFR mutation/amplification, 20 months, ongoing at data cut-off); alpelisib (CNS, PIK3CA mutation, 20 months, ongoing at data cut-off); panitumumab (head and neck, EGFR amplification, 20 months, progress); and trametinib (salivary gland, non-V600E BRAF mutation + FBXW7 mutation, progress). On average, patients in this subgroup of exceptionally long responders received matched targeted therapy for 19 months with six patients experiencing continuous clinical benefit at time of data cut-off (as indicated by arrowhead in Figure 4A). Within this group, only four recommendations were of tier 1 clinical evidence level (M1A), while the majority were of tier 2 clinical evidence level (5x M2A, 1x M2C). Of note and as outlined before, this group also included a recommendation with very low clinical evidence level M3. In addition to eleven patients with exceptionally long responses, nine (20.5%) patients experienced a clinical benefit on matched therapy for 6 to 11 months. Of note, within this group, a biliary duct cancer patient with BAP1 displayed a continuous response to olaparib at the end of data acquisition. The proportion of tier 1 level evidence recommendations in this group of responders was 55.6% (5/9). Another 21 patients were less than 6 months on a molecularly matched treatment regimen. Within this group, only four recommendations were of tier 1 clinical evidence level (1x PIK3CA/KRAS-mutated breast cancer, 1x MET-mutated kidney cancer, 1x ALK fusion-positive NSCLC, and 1x BRCA2-mutated pancreas cancer). Alectinib treatment of the ALK fusion-positive NSCLC and cabozantinib treatment of the MET mutated kidney cancer were accompanied by intolerable side effects, while in the case of the BRCA2 mutant pancreas cancer and the PIK3CA/KRAS-mutated breast cancer cases, deteriorating health conditions resulted in olaparib or alpelisib treatment cessation, respectively. In addition to the two tier 1 recommendations, this group of very short responders comprised the highest number of recommendations with low clinical evidence level (5x equal to or below M2C evidence level). 

In addition to patients receiving therapy based on CPG-informed MTB recommendations, follow-up data were also available for 22 patients without molecularly-matched treatment recommendations who underwent immune checkpoint inhibitor (ICI) therapy on the basis of particular molecular findings (MSI-H, MMR status, TMB high) or because of a positive PD-L1 expression status detected in routine immunohistochemical diagnostics. These patients received ICI for a median time of 3.3 months (min: 0.5 months; max: 27 months at data cut-off), of which eleven (50%) patients were on treatment for 3 months or longer (Figure 4B). Exceptional responses to ICI therapy were observed for two patients. A microsatellite instable (MSI-H)/mismatch repair-deficient (dMMR) colorectal cancer patient had a complete response after approx. 10 months on nivolumab, and an MSI-H/dMMR-mutated endometrial carcinoma patient exhibited an ongoing response to pembrolizumab at the time of data cut-off after already 27 months on ICI therapy (Figure 4B). Likewise, two lung cancer patients with high CPS values of 80 and 100 experienced a relatively long clinical benefit of an ICI therapy with pembrolizumab until progress or death for 14.3 or 12.5 months, respectively. This analysis reveals that, in addition to the provision of molecularly informed matched treatment recommendations, the assessment of MMR gene status, TMB, and PD-L1 expression levels represents another diagnostic procedure that may add additional therapeutical benefit to at least a subgroup of cases in the context of the MTB and, therefore, will be included in future MTB assessments.

## 4. Discussion

This retrospective study elucidates the diagnostic potential of NGS-mediated CGP for clinical decision making in a real-world setting of an interdisciplinary Molecular Tumor Board, with a particular focus on factors affecting clinical implementation. Between the years 2016 and 2020, the sequencing data of 554 cancer patients were discussed at the interdisciplinary MTB, of which clinical follow-up data were available in 332 cases. NGS-mediated CGP resulted in 41.9% (*n* = 139) of these cases in a molecularly informed therapy recommendation. Compliance with MTB therapy recommendation was observed in 44 (31.7%) of these 139 cases. These numbers are in line with results of comparable previous studies in which the proportion of CGP-informed MTB therapeutic recommendations ranged between 39.3 and 88% of all cases (median 54.1%) with a median implementation rate of 22.6% (min: 16.2%, max: 31.8%) of cases with MTB recommendations [6,8,9,10,12,14,33]. The comparably high compliance rate of the present study emphasizes, in particular, the quality and therapeutical relevance of CGP-informed MTB recommendations, and demonstrates the capability and feasibility to translate therapeutic recommendations into the clinic at the present institution, as well as at associated external medical centers. In fact, a significant clinical benefit, measured as time to treatment failure, was seen in almost half of patients receiving the MTB-recommended treatment modality. Here, in 45.4% (*n* = 20/44) of cases, the MTB-recommended treatment was applied for 6 months or longer until the earliest adverse event, i.e., progression, side effects, or death of patient. Of note, we also observed 13 cases in which the implementation of the recommended matched treatment failed due to an informed refusal by the patient or because the health care provider declined reimbursement of therapy costs. In eight of these cases, the recommended treatment was of high clinical evidence level (M1A: *n* = 2 and M2A: 6) with proven significant clinical benefit in the same or a different entity, suggesting that the implementation of these treatments would have further elevated the overall clinical benefit in our study cohort. Taken together, the relatively high compliance rate to MTB recommendations and the elevated proportion of therapeutic benefit emphasizes the strong clinical utility of the MTB at the Comprehensive Cancer Center Erlangen. Nevertheless, we also observed a significant proportion of non-adherence to MTB recommendations (68.3% of cases). The main reasons for the failure of clinical implementation were either the application of another molecularly unmatched treatment modality or deteriorating health condition/death of patient at time of MTB discussion. Indeed, a patient’s poor overall condition/death are among the main reasons for non-adherence to a CGP-informed MTB treatment recommendation throughout the literature [9,14,34]. For instance, Hoefflin et al. reported that worsening patient condition or the death of patient precluded MTB recommendation implementation in 22.9% or 18.6% of cases, respectively [10]. Similarly, Schwaederle et al. observed the death of 16% of patients before MTB recommendation implementation [8]. This exemplifies that, for a significant proportion of patients, molecular profiling at an earlier stage of disease is advisable. However, sudden unexpected health deterioration is commonly observed in the context of malignant progression, and the ad hoc identification of patients with a need for a fast-track molecular screening analysis is therefore challenging. Considering the implementation of NGS-mediated CGP at time of initial diagnosis in general may represent a potential solution circumventing this issue. However, this is not equally applicable to all cancer entities, given the scarcity of predictive biomarkers in some tumors, such as squamous cell lung cancer, gastric cancer, pancreatic cancer, or hepatocellular carcinoma [35]. Here, a targeted, single-gene analysis or the utilization of a small panel with selected genes frequently mutated in the respective entity is advisable. 

In more than half of cases with implementation of an unmatched therapy, MTB recommendations were of low clinical evidence level, implying inferior therapeutic potential which may have discouraged compliance with MTB recommendation. This is similar to the observation by Scheiter et al., who reported preferential therapeutical implementation of high clinical evidence recommendations, i.e., recommendations based on biomarkers predictive of a clinical response in the same entity [13]. In fact, in the present study, 27.3% of all recommendations with clinical translation were of M1A clinical evidence level, whereas only 13.7% M1A recommendations were found in the subgroup of cases with non-adherence to MTB recommendations. In contrast, the proportion of recommendations with very low clinical evidence level (i.e., M3 or below) was approximately 5 times higher in the subgroup of patients without clinical translation of MTB recommendations (21%) compared to the subgroup of patients who received the molecular matched therapy (4.6%). This indicates treatment recommendations of high clinical evidence level are preferentially implemented. Of note, on individual case level, we also observed the implementation of MTB recommendations with low clinical evidence level (M2C or lower). In fact, almost half (*n* = 4/10) of cases with an MTB recommendation of low clinical evidence level, i.e., M2B or below, exhibited a significant time to treatment failure of 9 months or longer, illustrating the limitations of interpreting the therapeutical potential of molecularly informed treatment recommendations solely on the grounds of a clinical evidence level score. In addition, other factors, such as driver mutation status, reciprocal effect of concurrent mutations, or level of gene copy number variation on the molecular level, as well as patient performance status, or number and type of previous therapies, need to be accounted for in the clinical assessment of molecularly guided therapy decision making. And in fact, this information is channeled adequately in an interdisciplinary Molecular Tumor Board. 

This study has limitations. First, this study is of a retrospective nature and lacks an unbiased control group for conclusively determining the clinical benefit of compliance to the molecularly informed MTB recommendation. Second, the study cohort consists of a heterogenous patient population in which some cancers were underrepresented. A subgroup analysis is therefore difficult and restricted to entities with sufficient case numbers. For instance, compared to the global incidence rates of breast and lung cancer [36], these malignancies appeared underrepresented in our study cohort. This may be, at least in part, attributed to already implemented routine molecular diagnostics tests (i.e., companion diagnostics), e.g., BRCA1/2, PIK3CA, or ERBB2/HER2 mutation screening for breast cancers and the introduction of a lung cancer-specific gene panel on site in 2018 [37], which facilitates the detection of therapeutically relevant alterations in these entities before patient inclusion in the MTB becomes necessary. The assessment of complex biomarkers, such as homologous repair deficiency (HRD), microsatellite analysis, and/or tumor mutational burden (TMB) was not part of the applied panel-mediated CPG. HRD is commonly observed in breast, ovarian, prostate, and pancreas cancer [38]. Since the latter two cancers constituted the major patient subgroups in our study cohort, and MTB case numbers for these cancers are still increasing, the implementation of routine HRD testing procedures, in addition to TMB and MSI assessment, is being considered at the MTB Erlangen.

## 5. Conclusions

The present study describes the successful implementation of a multi-disciplinary Molecular Tumor Board in clinical decision making. In particular, this study elucidates the factors and determinants impacting the clinical translation of a molecularly informed treatment recommendation which may guide the process of clinical decision making in future studies.

## Figures and Tables

**Table 1:** Cohort characteristics.

Characteristics		Value
Period		May 2016–December 2020
All cases	N (%)	590 (100)
Internal cases	N (%)	489 (82.9)
External cases	N (%)	101 (17.1)
Time diagnosis to MTB inclusion (months)		
All cases	median (Min, Max)	19 (0, 299)
Females	median (Min, Max)	15 (0, 299)
Males	median (Min, Max)	19 (0, 286)
Disease stage		
Local		N (% of All cases)
Relapse	N (% of All cases)	61 (10.3)
Metastasis	N (% of All cases)	410 (69.5)
Previous therapies		
Yes	N (% of All cases)	490 (83.1)
No	N (% of All cases)	29 (4.9)
Not evaluable	N (% of All cases)	71 (12.0)
Yes, females	N (% of All males)	193 (85.4)
Yes, males	N (% of All females)	297 (90.3)
Evaluable cases		
Total	N (% of All cases)	554 (93.9)
Females	N (%)	226 (40.8)
Median age	Yrs (Min, Max)	57 (16, 88)
Males	N (%)	329 (59.4)
Median age	Yrs (Min, Max)	63 (19, 88)
Localization primary tumor		
Colorectal	N (% Evaluable Cases)	87 (15.7)
Neuroendocrine	N (% Evaluable Cases)	53 (9.6)
Lung, NSCLC	N (% Evaluable Cases)	44 (7.9)
Pancreas	N (% Evaluable Cases)	44 (7.9)
Head and neck	N (% Evaluable Cases)	38 (6.9)
Soft tissue	N (% Evaluable Cases)	31 (5.6)
Prostate	N (% Evaluable Cases)	29 (5.2)
CUP	N (% Evaluable Cases)	26 (4.7)
Esophagus	N (% Evaluable Cases)	24 (4.3)
Biliary duct	N (% Evaluable Cases)	24 (4.3)
Gynecological	N (% Evaluable Cases)	22 (4.0)
Breast	N (% Evaluable Cases)	21 (3.8)
CNS	N (% Evaluable Cases)	19 (3.4)
Stomach	N (% Evaluable Cases)	18 (3.2)
Salivary gland	N (% Evaluable Cases)	14 (2.5)
Bladder	N (% Evaluable Cases)	11 (2.0)
Gallbladder	N (% Evaluable Cases)	11 (2.0)
Skin, non-melanoma	N (% Evaluable Cases)	9 (1.6)
Kidney	N (% Evaluable Cases)	7 (1.3)
Thyroid	N (% Evaluable Cases)	6 (1.1)
Small intestinal	N (% Evaluable Cases)	4 (0.7)
Germ cell	N (% Evaluable Cases)	3 (0.5)
Skin, melanoma	N (% Evaluable Cases)	3 (0.5)
Anus	N (% Evaluable Cases)	3 (0.5)
Hematopoietic	N (% Evaluable Cases)	2 (0.4)
Bone	N (% Evaluable Cases)	2 (0.4)
Thymus	N (% Evaluable Cases)	1 (0.2)

**Table 2:** Reasons for failure of clinical implementation of MTB recommendation dichotomized by entity.

		Clinical Translation:		Reasons for Non-Adherence (N):							
	MTB Recommend ^1^	Yes N (%)	No N (%)	OT	SD	PC	PD	NHR	PIR	DU	NA
**stomach**	3	0	3 (100)	2	-	1	-	-	-	-	-
**gynecological**	5	0	5 (100)	2	1	1	-	-	-	1	-
**pancreas**	8	2 (25)	6 (75)	3	-	-	-	-	1	-	2
**CUP**	4	1 (25)	3 (75)	-	2	-	-	-	1	-	-
**CNS**	11	3 (27)	8 (73)	3	2	1	1	-	1	-	3
**lung, NSCLC**	15	4 (27)	11 (73)	2	1	5	-	-	-	-	-
**colorectal**	17	5 (29)	12 (71)	3	-	5	2	2	-	-	-
**head and neck**	10	3 (30)	7 (70)	2	-	2	-	2	-	-	1
**neuroendocrine**	6	2 (33)	4 (67)	2	-	1	-	-	-	1	-
**esophagus**	9	3 (33)	6 (67)	-	-	1	2	1	-	1	1
**biliary duct**	8	3 (38)	5 (62)	-	-	3	-	1	1	-	-
**prostate**	5	2 (40)	3 (60)	2	1	-	-	-	-	-	-
**breast**	7	3 (43)	4 (57)	3	-	-	1	-	-	-	-
**soft tissue**	9	5 (56)	4 (44)	3	-	-	-	-	1	-	-
**esophagus**	9	3 (33)	6 (67)	-	-	1	2	1	-	1	1

^1^ total number of MTB recommendations for this entity. Abbreviations: OT = other therapy; SD = stable disease; PC = poor condition; PD = patient deceased; NHR = no healthcare reimbursement; PIR = patient informed refusal; DU = drug unavailable; NA = no data available.

**Table 3:** Summary of cases with clinical implementation of MTB recommendation.

Patient	Entity	Clinical Evidence Level	Drug Class/Inhibitor	Targetable Alteration	TTF (Months)
UKER28 UKER22 UKER62 UKER64 UKER253 UKER335 UKER336 UKER363 UKER361 UKER415 UKER440 UKER462 UKER68 *UKER72* UKER73 UKER161 UKER125 UKER86 UKER589 UKER398 UKER391 UKER185 UKER222 UKER231 UKER262 UKER458 UKER488 UKER490 UKER519 UKER533 UKER513 UKER565 UKER29 *UKER373* *UKER309* UKER61 UKER122 UKER166 UKER298 UKER502 *UKER504* UKER523 UKER233 UKER514	biliary duct biliary duct breast breast kidney lung, NSCLC lung, NSCLC lung, NSCLC lung, NSCLC pancreas pancreas prostate CNS *CNS* CNS colorectal colorectal colorectal CUP esophagus esophagus germ cell head and neck head and neck melanoma prostate salivary gland salivary gland soft tissue soft tissue soft tissue thyroid biliary duct *esophagus* *neuroendocrine* breast colorectal colorectal neuroendocrine skin, non-melanoma *skin, non-melanoma* soft tissue head and neck soft tissue	M1A M1A M1A M1A M1A M1A M1A M1A M1A M1A M1A M1A M2A *M2A* M2A M2A M2A M2A M2A M2A M2A M2A M2A M2A M2A M2A M2A M2A M2A M2A M2A M2A M2B *M2B* *M2B* M2C M2C M2C M2C M2C *M2C* M2C M3 M3	Infigratinib, clinical study Ivosidenib PARP inhibitor Alpelisib Cabozantinib Alectinib Crizotinib Dabrafenib/Trametinib Alectinib Olaparib Olaparib Olaparib Osimertinib *Dabrafenib/Trametinib* Alpelisib Crizotinib Olaparib Rucaparib Trastuzumab Osimertinib Olaparib Vemurafenib Panitumumab Paclitaxel/Trastuzumab Imatinib Olaparib Trametinib Trastuzumab Emtansine Crizotinib Olaparib Olaparib, after 3 mo. + Pembrolizumab Dabrafenib/Trametinib Olaparib Alectinib *Everolimus* Ponatinib Palbociclib Panitumumab/Trametinib Olaparib Palbociclib *Palbociclib* Everolimus Olaparib Ponatinib	PDE3B::FGFR2 gene fusion IDH1 p.Arg132Cys BRCA2 p.Asn3124Ile PIK3CA p.His1047Arg MET p.Met1268Thr EML4::ALK gene fusion MET GCN: 21.2 BRAF p.Val600Glu EML4::ALK gene fusion BRCA2 p.Cys3222Trpfs BRCA2 p.Tyr1894Ter BRCA2 p.Asn3124Ile EGFR p.Leu62Arg/p.Thr263Pro/ EGFR GCN: 33.4 *BRAF p.Gly466Glu/NRAS p.Gly12Asp* PIK3CA p.Cys420Arg MET GCN: 11.4 BRCA1 p.Ser4LeufsTer18 BRCA2 p.Ser3366AsnfsTer5 ERBB2 p.Arg678Gln/ERBB2 GCN: 4.9 EGFR p.Gly719Ala/EGFR GCN: 19 Franconia Anemia (FA)/ CCND1 GCN: 22.8/CDK4 GCN: 5.5/CCNE1 GCN: 5.2 BRAF p.Val600Glu EGFR GCN: 9.3 ERBB2 GCN: 6.5 KIT p.Ala502_Tyr503dup NBEA::BRCA2 gene fusion BRAF p.Asp594Asn/ FBXW7 p.Val464Met ERBB3 GCN: 3.7 + ERBB2 GCN: 3.2 TNS1::ALK gene fusion BRCA2 deletion BRCA2 p.Asn3124Ile BRAF p.Val600Glu BAP1 p.Ser37ArgfsTer47 BRE::ALK gene fusion *TSC2 p.Leu234SerfsTer60* FGFR1 GCN: 7.5/FGF3 GCN: 5.3/ FGF19 GCN: 4.8/FGF4 GCN: 4.5 CCND1 GCN: 10.3 MAP2K1 p.Lys57Glu ATM Splice Acceptor/ NBN p.Lys219AsnfsTer16 CDKN2A p.Asp84Val *CDKN2A p.Val82ArgfsTer44/* *CDKN2A p.Arg80Ter* PTEN p.Met134del FANCL p.Tyr111Cys FGFR4 GCN: 4.5	11 11 8 5 4 1 11 24 20 38 1 8 3 *0* 19 5 1 4 7 20 13 3 14 5 1 2 13 1 1 1 14 8 9 1 *0* 11 1 22 1 1 0 1 12 1

Shown is the data for the identified targetable alteration, the clinical evidence level of the applied matched treatment and the time to treatment failure (TTF) in months. Cases in *italic font* highlight cases with a duration of treatment below one month.

## Data Availability

The data for this study are available on request.

## Supplementary Materials

The following supporting information can be downloaded at https://www.mdpi.com/article/10.3390/cancers15245892/s1, Figure S1: Diagnostic capabilities of NGS-mediated CGP in tumor origin re-classification.; Figure S2: Molecular alterations identified by gene panel-mediated CGP.; Table S1: RNA alterations identified by NGS-mediated CGP and FISH.; Table S2: Summary of non-implemented MTB recommendations.
