# Supplementary material for: Determinants Affecting the Clinical Implementation of a Molecularly Informed Molecular Tumor Board Recommendation: Experience from a Tertiary Cancer Center
Source: Cancers (Basel). 2023 Dec 18;15(24):5892. doi: 10.3390/cancers15245892 (PMC10741918; doi:10.3390/cancers15245892)
Supplement: Supplementary file 1 [file cancers-15-05892-s001.zip › Supplementary_Figures.pdf]

Supplementary Figures

Figure S1

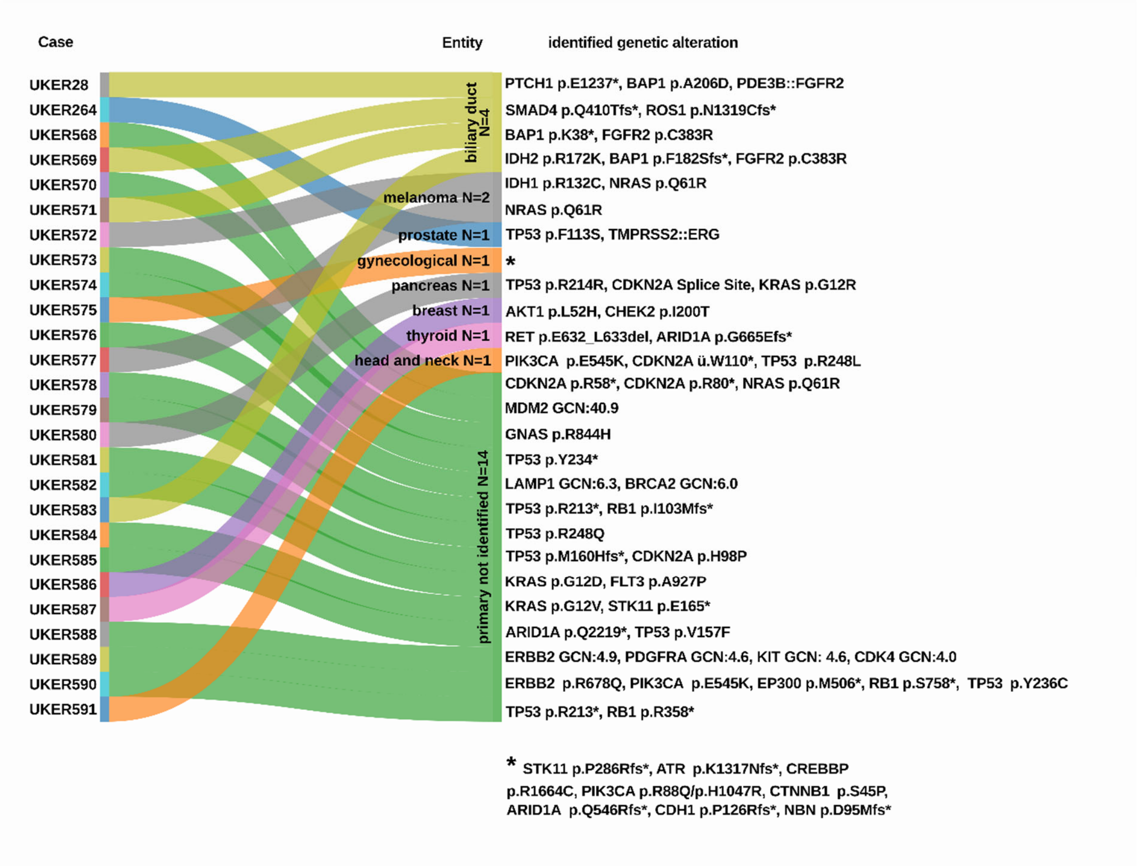

Figure S1. Diagnostic capabilities of NGS mediated CGP in tumor origin re-classification. The tissue of cancer origin was unknown in 26 cancer cases (cancer of unknown primary, CUP). NGS mediated CGP facilitated primary identification in 12 cases, whereas no information on site of tumor origin was inferable from CGP for 13 cancer cases.

Figure S2

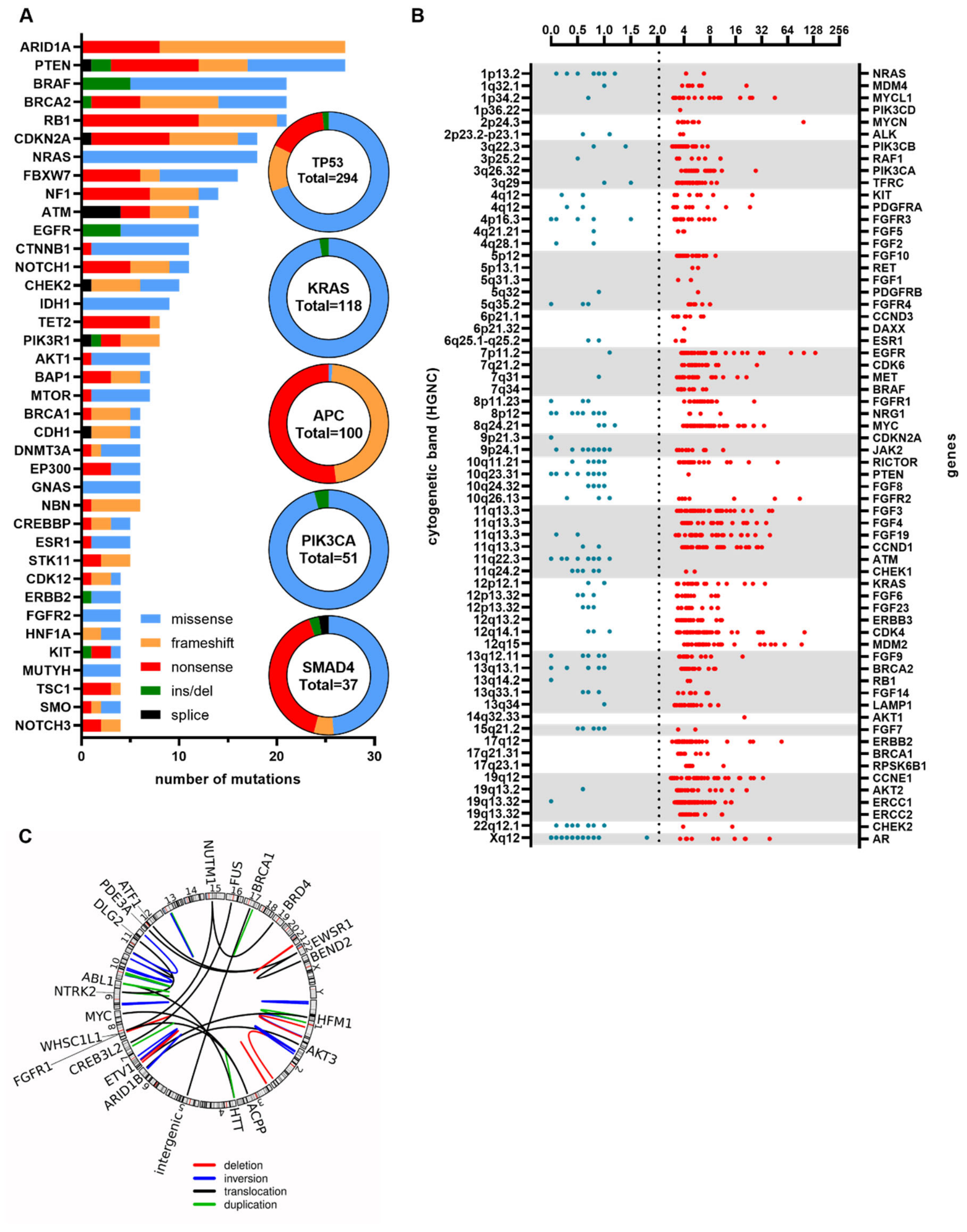

Figure S2. Molecular alterations identified by gene panel mediated CGP. (A) Types of mutations found in recurrently altered genes. Shown are the fraction of missense, frameshift and nonsense mutations, as well as, short insertions/deletions (ins/del) and splice site alterations. The distribution of different genomic alterations of the five most frequently mutated genes are summarized in donut charts. (B) Copy number variants (CNV) of analyzed genes. Computed gene copy numbers (GCN) are blotted as deletions (GCN < 2.0; blue dots) and amplifications (GCN > 2.0; red dots). The dotted line indicates a normal, bi-allelic CNV (GCN: 2.0). (C) Overview of detected gene fusions. Circos blot shows intrachromosomal translocations (black), as well as intrachromosomal deletions (red), duplications (blue), and inversions (green). For simplicity, only genes involved in translocations are shown.
